# How direct healthcare professional communications are operationalised by general practitioners and community pharmacists in Ireland: a national cross sectional study

**DOI:** 10.1007/s11096-026-02105-3

**Published:** 2026-03-11

**Authors:** Paul E. Ryan, Ann Sinéad Doherty, Darren L. Dahly, Stephen Byrne, Sinead Curran, Darren Scully, Ruchika Sharma, Emma Wallace

**Affiliations:** 1 Department of General Practice, School of Medicine, University College Cork, Cork, Ireland; 2 HRB Clinical Research Facility, University College Cork, Cork, Ireland; 3 School of Public Health, University College Cork, Cork, Ireland; 4 School of Pharmacy, University College Cork, Cork, Ireland; 5 Health Products Regulatory Agency, Dublin, Ireland

**Keywords:** Community pharmacy, Medication safety, Regulatory communications

## Abstract

**Introduction:**

Direct Healthcare Professional Communications (DHPCs) alert healthcare professionals of important safety information relating to medication(s) and of the need to adapt practices with respect to these. International evidence suggests that their implementation varies in clinical practice. To date, few studies have examined implementation of DHPCs in primary care.

**Aim:**

To examine how general practitioners (GPs) and community pharmacists implement DHPCs in Ireland and their preferences for receiving medication safety updates.

**Method:**

A national cross-sectional survey of GPs and pharmacists in collaboration with the Irish Health Products Regulatory Authority (HPRA), was conducted in June 2024. GPs and CPs were invited to participate via national gatekeepers (Irish College of GPs, Pharmaceutical Society of Ireland). Following piloting, the questionnaire were administered via email using Qualtrics. Data was analysed using R and R Studio.

**Results:**

A total of 277 GPs and 219 pharmacists completed the questionnaire, a response rate of 6% and 8% respectively. Most GPs (n = 227, 82%) and pharmacists (n = 196, 89%) reported DHPCs as their preferred source of medication safety updates. Practice protocols for sharing DPHCs once received differed across the two professional groups. For example, DHPCs were more likely to be disseminated and discussed at a pharmacy practice meeting (n = 64 pharmacists, 29%) compared with GP practice meetings (n = 24 GPs, 9%). More than one-third of GPs (n = 98, 35%) identified time constraints as the most important barrier to DHPC implementation, followed by absence of prescribing notifications on patient electronic health records (EHRs), n = 36 GPs (13%), n = 39 CPs (18%). A total of 257 GPs (93%) and 198 CPs (90%) identified patient EHR prescribing alerts, aligned with DHPC recommendations, integrated at the point of patient care as a preferred way to support implementation.

**Conclusion:**

Surveyed GPs and pharmacists use DHPCs as their primary information source for new medication safety alerts and most reported these communications as very useful. Repeated DHPC communications across different modalities were valued. Barriers to implementation included time constraints and lack of point of care alerts for both GPs and pharmacists. Remote clinical support is acceptable to GPs and pharmacists and may support the implementation of DHPC recommendations to optimise medication safety in primary care.

**Supplementary Information:**

The online version contains supplementary material available at 10.1007/s11096-026-02105-3.

## Impact statements

- Integrating prescribing alerts aligned with DHPC recommendations into the patient electronic healthcare record at the point of care has the potential to support DHPC implementation.
- Clarifying responsibility for DHPC related actions across both primary and secondary care may support DHPC implementation
- Most community pharmacists and GPs were willing to engage with remote clinical support indicating collaborative medication review systems could enhance DHPC implementation in busy primary care environments.

## Introduction

Direct healthcare professional communications (DHPCs) ‘*communicate important safety information directly to individual healthcare professionals by a marketing authorisation holder or a competent authority, informing them of the need to take certain actions or adapt their practices in relation to a medicinal product’* [1].

The marketing authorisation holder is the company or other legal entity that has the authorisation to market a medicine in a country. In Ireland, the Health Products Regulatory Authority (HPRA) is responsible for monitoring medication safety. One role of the HPRA and other competent authorities (e.g. European Medicines Agency (EMA)), is to monitor emerging safety data, including previously unrecognised adverse drug reactions (ADRs). Competent authorities also monitor for any change in the characteristics of known ADRs, such as frequency or severity. Competent authorities may then direct marketing authorisation holders to distribute DHPCs on new important safety issues to optimise patient safety. In Ireland, DHPCs are typically disseminated to healthcare professionals by letter.

There is mixed evidence on the effectiveness of DHPCs globally. A 2019 systematic review with meta-analysis (n = 40) examined the impact of 25 different regulatory risk communications such DHPCs and drug bulletins in the UK [2]. This review found medication risk communications significantly influence prescribing, with DHPCs associated with greater relative prescribing mean changes (−47%) than drug bulletins (−13%). However, these reductions were largely driven by the larger mean reduction observed (−78%) for DHPCs regarding product withdrawals, with smaller mean reductions observed for other DHPC categories including those involving restrictions or changes in indication with recommendations for action (−34%). A 2020 systematic review included 72 studies between 1998 and 2018 and examined the impact of different medication safety warnings on intended changes in medication prescribing, including DHPCs. The review found 28/72 (39%) of studies were effective, 12/72 (17%) were not effective and 26/72 (36%) described a partial implementation of the warnings [3]. In 2020, a US FDA drug safety communication on the risk of non-melanoma skin cancer with hydrochlorothiazide was only associated with a clinically negligible change in the monthly prescribing of hydrochlorothiazide drugs (0.018%) over a two year period [4].

A recent review (2025) examined 60 studies that evaluated the impact of 31 European DHPCs between 2008 and 2022 [5]. As DHPCs differ in their content and aim, this review examined a range of desired outcomes related to each DHPC including prescribing trends, ADR knowledge/awareness, health outcomes, monitoring and self-reported behaviour (prescribing/monitoring). For example, for DHPCs advising dose changes the desired outcome was prescribing at that particular dosage. Less than one-third (29%) of the 103 desired outcomes (e.g. prescribing, knowledge) were impacted to a high degree by DHPCs, with almost one-quarter (23%) showing limited or no impact [5].

In line with the mixed evidence of DHPCs in changing desired outcomes such as prescribing behaviour, these communications may need repeating [6]. A recent EMA-funded study suggests that, despite safety-related restrictions on fluoroquinolone prescribing introduced in 2019, these antibiotics may still have been prescribed outside their clinical indications until 2021 [7]. Consequently, a second DHPC was issued in June 2023 to remind healthcare professionals (HCPs) of their risks and to reinforce the existing restrictions regarding clinical use [6]. The mixed evidence in DHPC effectiveness likely stems from variation in both the DHPCs themselves and the contexts in which they are implemented.

This mixed evidence regarding DHPC impact on outcomes of interest could be due to practitioner factors, warranting further investigation. There is limited research to date focused on primary care professionals [8–10], where most prescribing takes place [11]. Understanding how primary care professionals manage and operationalise DHPCs in clinical practice will help to identify barriers and enablers in DHPC implementation.

### Aim

To examine how general practitioners (GPs) and community pharmacists implement DHPCs in Ireland and their preferences for receiving medication safety updates.

## Method

### Survey design

A national cross-sectional study was conducted between May and June 2024, with de-novo survey questions aligned with survey objectives developed in collaboration with the pharmacovigilance team of the HPRA who reviewed and commented on the questions. The Strengthening the Reporting of Observational Studies in Epidemiology (STROBE) Statement was used to guide reporting of this study [12]. The questionnaire collected data on:

1. GP/community pharmacist demographics (e.g. age, sex, years in practice)
2. Medication safety update experiences, beliefs and perceptions (e.g. how DHPCs are received, knowledge and experience of DHPCs and medication safety information preference)
3. Practical implementation of DHPCs (e.g. operationalisation of DHPCs including barriers and enablers to implementation in practice).

The questionnaire (see supplementary material) was piloted on four practicing GPs and four community pharmacists for clarity of questionnaire items and time taken to complete. Suggested changes were incorporated into the questionnaire following piloting e.g. allowing multiple responses for some questions. Questionnaire responses were collected online using Qualtrics XM (Qualtrics 2024).

### Participant recruitment and data collection

GPs and community pharmacists (hereafter referred to as pharmacists) registered to practice in Ireland were eligible to participate. The national GP professional body in Ireland (Irish College of General Practitioners (ICGP)) acted as a gatekeeper and disseminated the questionnaire to its members via their continuing medical education network in May 2024. In addition, 834 GPs who attended an ICGP national education webinar on 1st May 2024 were sent a link to the questionnaire via the chat function during the webinar. There were 4,456 GPs registered as members of the ICGP in 2023 [13]. For pharmacists, the national pharmacy regulator, the Pharmaceutical Society of Ireland (PSI), acted as gatekeeper to distribute the questionnaire by providing access to their email database (n = 3965 community pharmacists who consented to research invitations). A link to the questionnaire was emailed in May 2024 with a reminder email sent after two weeks. The questionnaire was open for six weeks in total. No incentives were provided for participation.

### Data analysis

Anonymous questionnaire data were exported to Microsoft Excel©. Data were reported as percentages and frequencies. Free text responses (for ‘*other’* and open-ended questions) were analysed looking for frequently occurring terms and text.

Quantitative questionnaire responses were explored using appropriate descriptive statistics and analysed using logistic regression. Quantitative analyses were conducted using R (version 4.4.2 [14] and RStudio (version 2024.12.1-563) using the gtsummary [15] and ggplot2 [16] packages.

### Ethics approval

Ethical approval for this study was granted on 12/3/24 by the Irish College of General Practitioners (ICGP) Research Ethics Committee (reference number 2083).

## Results

### Participant demographics

A total of 277 GPs completed the questionnaire, representing 6% of GPs registered with the ICGP on the date the questionnaire was administered. This response rate is lower than previous national ICGP surveys (approximately 14% response rate) [17]. A total of 219 community pharmacists completed the questionnaire, representing 6% of community pharmacists who consented to be contacted for research purposes (n = 3965), 4% of the national register of community pharmacists (n = 5382) and 3% of the national register of pharmacists (n = 7732) [18]. This response rate is similar to previous national community pharmacist studies (approximately 5% response rate) [19].

Of those who completed the questionnaire, a total of 82 (30%) GPs and 95 (43%) pharmacists had worked between 15 and 29 years in practice with 163 (59%) GPs and 90 (41%) pharmacists working 4–5 days per week in clinical practice. Fifty-five percent of GP respondents and 68% of pharmacist respondents were female which is representative of both the GP [20] and pharmacist [18] workforce. Further details on participant characteristics are presented in Table 1.

**Table 1:** Survey demographics of GP (N = 277) and community pharmacist (N = 219) participants

Characteristic	GPs N = 277	Community pharmacists N = 219
*Age group (years)*		
20–24	–	4 (2%)
25–34	9 (3%)	32 (15%)
35–44	104 (38%)	63 (29%)
45–54	84 (30%)	68 (31%)
55–64	52 (19%)	43 (20%)
65–74	28 (10%)	8 (4%)
75 and over	–	1 (1%)
*Gender*		
Female	153 (55%)	149 (68%)
Male	124 (45%)	69 (32%)
Non-binary	–	1 (1%)
*Primary workplace*		
General Practice/Pharmacy	265 (96%)	214 (98%)
Academia	4 (1%)	0 (0%)
Industry	0 (0%)	1 (1%)
*Pharmacy Practice type*		
Large chain (more than 10 pharmacies)	–	48 (22%)
Single independent pharmacy	–	98 (45%)
Small chain (2–10 pharmacies)	–	72 (33%)
*Years working in General Practice/Community Pharmacy since qualification*		
< 5	35 (13%)	20 (9%)
5–14	100 (36%)	58 (26%)
15–29	85 (31%)	93 (42%)
30 +	57 (21%)	48 (22%)
*Professional role*		
GP Principal/Superintendent Pharmacist	178 (64%)	55 (25%)
Assistant GP/Supervising Pharmacist	17 (6%)	64 (29%)
Locum GP/Pharmacist	5 (2%)	34 (16%)
Salaried GP/Support pharmacist	42 (15%)	66 (30%)
Sessional GP	18 (7%)	–
Single handed GP	12 (4%)	–
Other e.g. academic work	5 (2%)	–
*Number of days a week worked in GP/Community Pharmacy*		
1 day or less	9 (3%)	13 (6%)
2–4 days	90 (32%)	90 (41%)
4–5 days	163 (59%)	90 (41%)
> 5 days	13 (5%)	26 (12%)
Not working in clinical practice	2 (1%)	–
*Number of other GPs/Pharmacists working with you*		
0 (single handed GP/Pharmacist)	18 (7%)	70 (32%)
1–2	66 (24%)	101 (46%)
3–4	119 (43%)	40 (18%)
5 or more	72 (26%)	8 (4%)
Not working in Clinical Practice	2 (1%)	
*Daytime site of work*		
I work across multiple sites on a regular basis	40 (14%)	29 (13%)
I work in multiple sites on an irregular basis	7 (3%)	35 (16%)
I work in one site only	230 (83%)	155 (71%)
*Location of practice*		
Urban (town with a population > 1500 persons)	195 (70%)	150 (68%)
Rural (town with a population < 1500 persons)	50 (18%)	48 (22%)
Work in both a rural and urban location	31 (11%)	21 (10%)
Not working clinically	1 (1%)	

Other GPs: GPs in academia.

GP Principal: GP who is a partner or co-owner of a practice with responsibility for clinical care and the management of the practice.

Salaried GP: GP employed on a fixed salary within a General Practice.

Sessional GP: GP who works as a sessional or part-time basis within a General Practice.

Assistant GP: GP employed within a General Practice typically without holding partnership status.

### Preferences on methods of communication and receipt of DHPC communications

**Fig. 1:**
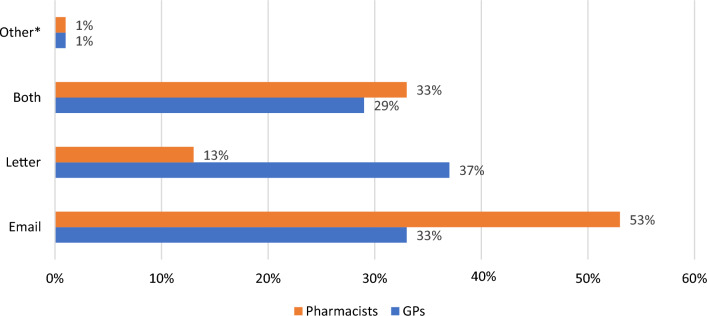
Preferred method of correspondence regarding important medication safety related information (n = 277 GPs, n = 219 community pharmacists). *Other (Community Pharmacists): Upload to correspondence section of the Primary Care Reimbursement Service (PCRS) suite. *Other (GPs): Email with supplementary audio/video summary of key points, flagged on software, healthlink

A higher proportion of pharmacists (n = 116, 53%) preferred to receive DHPCs via email compared with GPs (n = 92, 33%) (Fig. 1).

Most respondents reported that they currently receive DHPCs by letter, email or both forms of communication (Fig. S1).

**Fig. 2:**
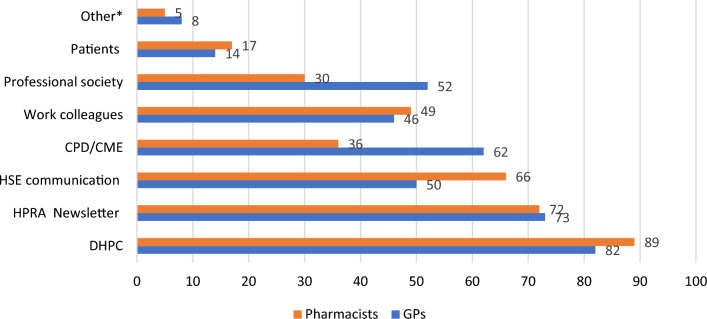
Primary source of information on important medication safety updates (n = 277 GPs, n = 219 Community Pharmacists). *Other (Community pharmacists) Pharmacy union, online platforms. *Other (GPs): Continuing Professional Development (CPD) companies, normal media (radio/TV), national medicines information centre, online searches

The majority of GPs (n = 227, 82%) and pharmacists (n = 196, 89%) used DHPCs as their primary information source for important medication safety updates (Fig. 2).

Regarding usefulness of DHPCs to support safe prescribing, one quarter (n = 70, 25%) of GPs and 35% (n = 76) of pharmacists found DHPCs very useful, while only 1% (n = 2) of pharmacists and 4% (n = 11) of GPs felt DHPCs were not at all useful (Fig. S2). The majority of GPs and pharmacists found repeated DHPC communications useful over time, with 30% (n = 82) GPs and 24% (n = 53) pharmacists finding it moderately useful and 25% (n = 70) GPs and 40% (n = 88) pharmacists finding it very useful (Fig. S3). Receiving medication safety information via multiple simultaneous methods was considered very helpful by 26% (n = 71) of GPs and 40% of pharmacists (n = 88) (Fig. S4).

### Actions undertaken when DHPC is received in practice

**Fig. 3:**
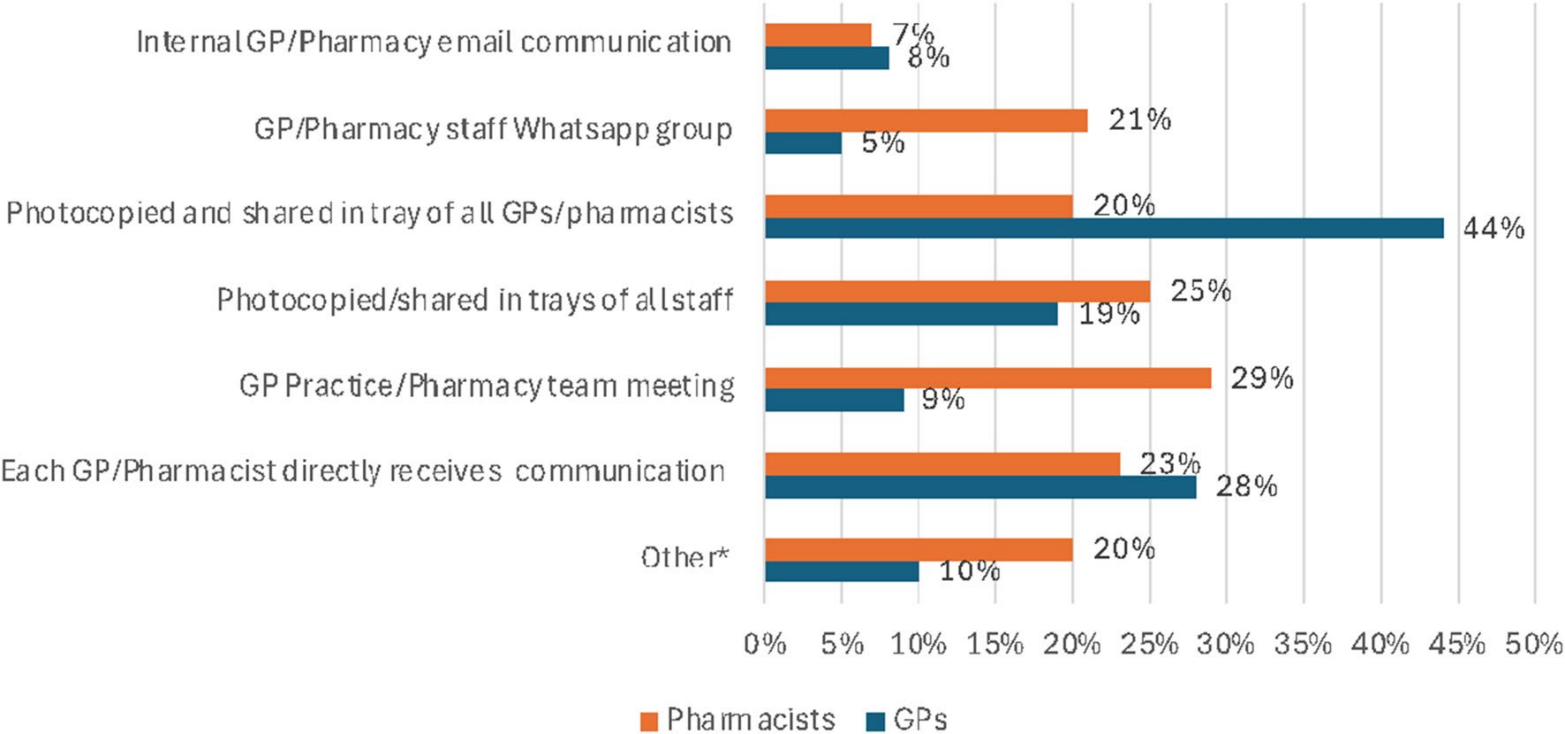
DHPC dissemination to GP practice/pharmacy staff (n = 277 GPs, n = 219 community pharmacists). *Other (Community pharmacists): handover diary, left in communal area, communications folder. *Other (GP) personal notice board, drug update folder/chart, ad hoc, left at reception, tasked to GPs

Practice protocols for sharing DPHCs following their receipt differed across both professional groups. For example, DHPCs were more likely to be disseminated and discussed at a pharmacy practice meeting (n = 64 pharmacists, 29%) compared with GP practice meetings (n = 24 GPs, 9%). DHPCs were photocopied and distributed via clinical practice staff post trays in general practice (n = 123, 44%) but this was less common in community pharmacy (n = 54, 25%) (Fig. 3).

**Fig. 4:**
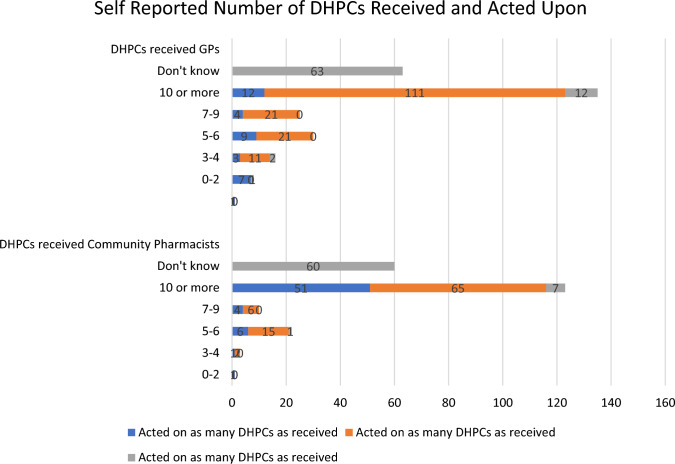
Comparison of self-reported recall of number of DHPCs received and the proportion acted upon in the last five years, by GPs (N = 277) and community pharmacists (N = 219)

GPs and pharmacist differed in their recall of DHPCs. Of 198 GPs who reported both receiving *and* acting on DHPCs, just 34 (17%) GPs reported acting on every DHPC received, compared with 63 of 151 pharmacists (42%). Based on logistic regression analysis, this equated to GPs having a significantly lower odds of acting on DHPCs (odds ratio [OR] = 0.29, 95% confidence interval [CI] 0.18–0.47; *p* < 0.001). (Table S1 and Fig. 4).

### Management of DHPCs that include recommendations

Seventy-two pharmacists (33%) indicated they contacted relevant patients to discuss DHPC prescribing recommendations, compared with 33 GPs (12%). GPs reported not contacting patients directly when they had already implemented the recommended prescribing change or when the recommendation related to short term (acute) rather than long-term prescriptions. A total of 33 GPs (12%) and 75 pharmacists (34%) put software notification alerts on relevant electronic patient medical records to discuss the DHPC recommendations at the next repeat prescription request (Fig. S5).

### Knowledge of and actions following DHPCs on fluoroquinolones (2023) and sodium valproate (2018)

The questionnaire included questions on DHPCs issued for fluoroquinolones and sodium valproate. Two hundred and seventy-two (98%) GPs and 217 pharmacists (99%) correctly identified teratogenicity as the primary concern associated with sodium valproate. Most GPs (n = 272, 87%) and pharmacists (n = 198, 90%) correctly identified musculoskeletal and nervous system adverse effects as the main safety issues associated with fluoroquinolones. Following receipt of the fluoroquinolone DHPC, 128 GPs (46%) reported increased awareness of their adverse effects profile, with 168 (61%) indicating they reduced their prescribing of these agents (Fig. S6). Almost three-quarters (n = 160, 73%) of pharmacists reported increased awareness of fluoroquinolones’ adverse effects, with 82 (37%) reporting discussion of these with patients at dispensing (Fig. S7).

Following receipt of the sodium valproate DHPC, 137 GPs (49%) reported increased awareness of adverse effects and were more likely to counsel patients on this, compared with 172 pharmacists (79%). GPs may have already been aware of the teratogenic risk as 272 (98%) GPs correctly identified teratogenicity as the primary concern with sodium valproate. Fewer GPs identified patients and put a note on their EHR following this sodium valproate DHPC (n = 83, 30%) compared with 110 (50%) of pharmacists (Fig. S8). In open-ended questions GPs reported a lack of clarity on whose responsibility it is to ensure informed consent is received when sodium valproate is initiated in secondary care.

### Barriers to and enablers for implementation of DHPCs in practice

**Fig. 5:**
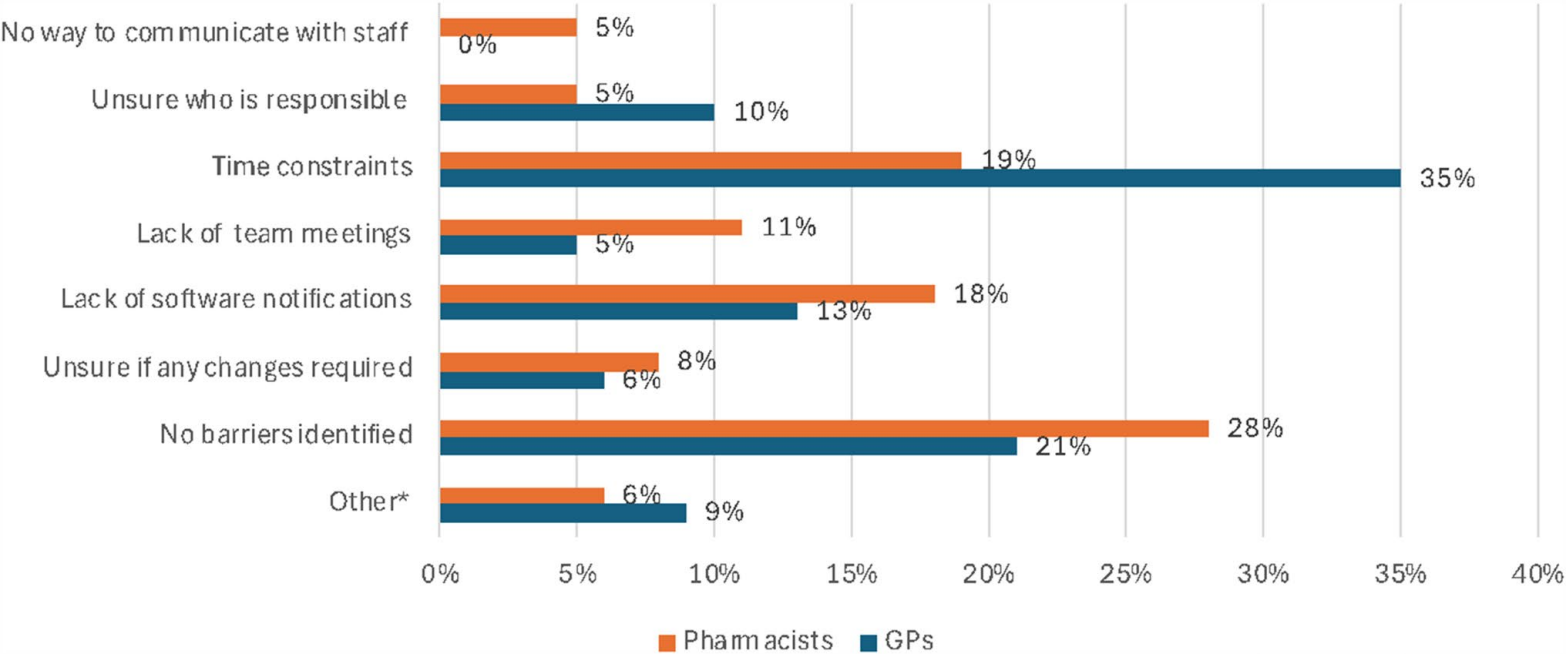
Barriers to the implementation of DHPC recommendations (n = 277 GPs, n = 219 community pharmacists). *Other (GPs): Receiving too many letters emails, not always relevant, overwhelming administrative workload. Other (Community Pharmacists): Not prioritised, not always relevant

Sixty-one (28%) pharmacists and 57 GPs (21%) identified no barriers to DHPC implementation in practice (Fig. 5).

Reported barriers included GP and pharmacist time constraints (n = 98 GPs, 35%; pharmacists: n = 41, 19%). Other barriers reported were a lack of prescribing notifications on patient EHRs (GPs: n = 36, 13%; pharmacists: n = 39, 18%), no practice team meetings to discuss DHPCs (GPs: n = 15 GPs, 5%; pharmacists: n = 24, 11%) and lack of clarity on overall clinical responsibility to enact DHPC recommendations (GPs: n = 28 GPs, 10%; pharmacists: n = 11, 5%). In an exploratory analysis using logistic regression with Bonferroni adjustment for multiple comparisons, we did not observe significant differences in the barriers or actions taken that respondents reported by urban/rural setting, years of experience, or pharmacy practice type. However, it is important to note that the study may not have been powered to reliably detect more modest differences. Several enablers for DHPC implementation were identified including prescribing alerts aligned with DHPC recommendations being integrated into the patient EHR at the point of care (GPs: n = 257, 93%; pharmacists: n = 198, 90%). A total of 230 GPs (83%) and 164 pharmacists (75%) indicated willingness to receive remote clinical support (e.g. pharmacist or GP) to help implement DHPC recommended changes in their practice.

## Discussion

### Principal findings

This national survey found that most pharmacists and GPs considered DHPCs useful and relied on them as a primary information source. Implementation methods differed at the practice level, with pharmacists reporting acting on a higher proportion of DHPCs received than GPs.

### Comparison with previous literature

In this study, most pharmacists and GPs indicated that DHPCs were their primary source for new medication safety information which contrasts with a recent study showing that guidelines, rather than DHPCs, were the preferred source of information on thrombosis risk with Covid vaccines [21].

Most surveyed pharmacists and GPs considered DHPCs a useful source of medicine information, a finding that contrasts with earlier qualitative work in Danish GPs [22]. However, that most GPs reported repeated DHPCs as useful aligns with previous European studies [9, 23].

Unlike the minority of GPs in this study who prefer DHPC communication via email alone, Dutch [24] and Danish [25] HCPs (studies examined a broad range of HCPs including GPs) indicated a preference for receiving medication safety updates via e-mail. A study in The Netherlands [26] informed the regulator’s approach there and since October 2024, DHPCs are primarily sent digitally to relevant HCPs with a known email address; otherwise a postal copy is issued [27]. However, it must be noted that Ireland currently has a mixed private public healthcare model with resultant less integrated communication and shared medication records compared with the universal healthcare systems present in the Netherlands and Denmark. This may explain the differences seen in DHPC communication preferences found in this study.

At practice level, pharmacists and GPs used different dissemination and implementation methods in line with their professional practice, with pharmacists more likely to discuss the DHPC at a practice meeting, and GPs more likely to share a photocopy of the letter/email with practice staff. This is similar to a previous study which demonstrated discussing medication safety issue with colleagues was the most common stated action [28].

Pharmacists and GPs act at different points in the patient journey due to their different roles and responsibilities. For example, GPs and pharmacists act at different points in the patient journey due to their different roles and responsibilities. For example, at the time of survey distribution pharmacists did not have prescribing rights, however recent legislative amendments in October 2025 enables pharmacists to prescribe in eight common conditions seen in primary care which may have implications for DHPC processes. As there is no shared patient electronic health record in Ireland currently, community pharmacists cannot currently access the complete electronic medication record which may also have implications for DHPC processes. As pharmacists are the last person to verify a medication before the patient receives it they are often referred to as the ‘gatekeeper’ [29] or ‘infallible backstop’ [30] by prescribers in qualitative studies. This may explain why DHPCs were reported to be more useful by pharmacists compared with GPs, with pharmacists more likely to contact relevant patients directly. When recalling DHPCs acted upon over the past five years, pharmacists were more likely than GPs to report acting on those received. As pharmacists in Ireland dispense medications from multiple settings (e.g. primary and secondary care) including multiple GPs, this may expose them to a broader case mix of patients and thus provide more opportunities to encounter medications subject to DHPCs, which may necessitate action.

Surveyed pharmacists and GPs demonstrated very high levels of understanding of DHPCs for sodium valproate and fluoroquinolones, in keeping with previous studies on awareness and knowledge of DHPCs [31, 32]. These particular DHPCs were selected as examples of prominent DHPCs relating to commonly prescribed medications in Ireland. Sodium valproate was chosen as it is included in the pregnancy prevention programme in Ireland and is considered a higher stakes medication due to its adverse effect profile. Sodium Valproate is specialist initiated In Ireland but repeat prescriptions are issued by GPs. Fluoroquinolones are an example of a medication that is more commonly prescribed by GPs and may be GP initiated. Almost two-thirds of GPs indicated they had subsequently reduced their fluoroquinolone prescribing, demonstrating the important medication safety role of DHPCs. Surveyed pharmacists were more responsive to the valproate DHPC although recall bias may have limited the accuracy of responses, as the valproate DHPC had been issued more than five years earlier, in 2018.

Time constraints were highlighted as the primary barrier to DHPC implementation by pharmacists and GPs which aligns with findings from a recent systematic review published on barriers to implementation of medicine risk communications [10]. When considering the barriers to DHPC implementation, whilst the medicine information in most DHPCs is urgent, other DHPCs may not be as urgent i.e. those DHPCs relating to medications that the GP or pharmacist may not be as familiar with. This potentially dilutes the impact of messages that require urgent attention. Respondents were not asked about varying responses to different DHPCs so results need to be interpreted with this in mind. The vast majority of pharmacists and GPs suggested that point of care medication safety alerts aligned with DHPC recommendations would support the implementation of recommendations in clinical practice, which is consistent with a previous study which reported that hospital-based pharmacists prefer to receive safety notifications at the point of prescribing [28]. Both professional groups indicated willingness to receive remote clinical support from another pharmacist or GP to support implementation of DHPC recommendations. This system is already available in some countries, such as the UK, where GP practice pharmacists remotely review medications and can support DHPC implementation [33].

The present study extends the literature on DHPCs as to the best of our knowledge no prior studies have examined their operationalisation once received into GP practices or community pharmacies. The nature of the specific DHPC received was not explored in this study and implementation actions may vary depending on the perceived seriousness of the DHPC message, although evidence for actioning based on level of seriousness is mixed [5, 28].

### Clinical, policy and research implications

One of the roles of medicines regulators is to ensure communication of DHPCs to target HCPs. Since the establishment of the EMA in 1995 there has been an increase in the number of new medications approved over the years, with 424 new medications being authorised in the 10 year period between 2013 and 2023 [34]. With the growing number of medications, the likelihood of previously unknown or less common adverse reactions emerging post-licensing increases. This study has shown that DHPCs are trusted and their information is applied in practice. The overall aim of DHPCs is to reduce medication-related harm, something which is estimated to cost the National Health Service in England £2.21 billion annually [35].

A six year review of medication related harm clinical litigation in Ireland between 2011 and 2016 found the median total cost of claims was €60 991, including median damages of €33 858. [36]. A more recent Irish cohort study involving older persons admitted to hospital found the average cost associated with hospital admission for an ADR was €9538 [37].

Understanding how DHPCs are implemented at a practice level, as well as the barriers to their implementation, can inform potential new approaches to their development and dissemination to improve HCP knowledge [38] and clinical practice [39]. Improved implementation may reduce medication-related harm, translate into fewer ADRs, fewer hospitalisation, and consequently improved patient care [40]. This study has informed of the actions taken following DHPC receipt and identified barriers and enablers to their implementation. Further research, including qualitative enquiry, is required to fully understand how the various barriers and enablers to their implementation may interact in practice. Standardised, tiered workflows in the pharmacy based on level of DHPC urgency combined with point-of-care alerts in pharmacy systems have potential to streamline DHPCs implementation and integration into routine practice. Proactive patient identification and counselling, supported by timely prescriber communication and remote clinical support, may have a role in strengthening the implementation of DHPC recommendations.

### Strengths and limitations

This is the first national survey on DHPC implementation in primary care which included two HCP groups. The study was designed in collaboration with the Irish medicines regulator, the HPRA. The questionnaire was developed de-novo based on the literature and piloted to optimise length and readability. Selection bias was minimised by ensuring GPs and pharmacists were contacted through the national professional body for GPs (ICGP) and the pharmacy regulator (PSI).

However the response rate was relatively low and so caution is required in generalising these findings to the national population. Low response rates are a feature of previous national surveys involving HCPs [41].

As with other questionnaires, recall bias may have occurred as responses relied on self-reporting which may have been influenced by recollection of past experiences. To mitigate this a more recent 2023 DHPC on fluoroquinolones was included.

## Conclusion

Surveyed GPs and pharmacists use DHPCs as their primary information source for new medication safety alerts and most reported these communications as very useful. Repeated DHPC communications across different modalities were valued. Barriers to implementation included time constraints and lack of point of care alerts for both GPs and pharmacists. Remote clinical support is acceptable to GPs and pharmacists and may support the implementation of DHPC recommendations to optimise medication safety in primary care.

## Supplementary Information

Below is the link to the electronic supplementary material.

Supplementary file1 (DOCX 52 KB)

Supplementary file2 (DOCX 306 KB)

## Data Availability

Survey data and analysis code is available at open science framework repository https://osf.io/aqk65/.
