# Supplementary material for: How direct healthcare professional communications are operationalised by general practitioners and community pharmacists in Ireland: a national cross sectional study
Source: Int J Clin Pharm. 2026 Mar 11;48(4):1303–14. doi: 10.1007/s11096-026-02105-3 (PMC13369712; doi:10.1007/s11096-026-02105-3)
Supplement: Supplementary file 1 — Supplementary file1 (DOCX 52 KB) [file 11096_2026_2105_MOESM1_ESM.docx]

**Supplementary Information**

**Fig. S1:** Mode of receipt of DHPCs in general practice/pharmacy (n=277 GPs, n=219 community pharmacists)

**Fig. S2**: Usefulness of DHPCs in relation to safe prescribing and dispensing (n=277 GPs, n=219 community pharmacists)

**Fig. S3**: Usefulness of repeated alerts regarding important medication safety related information (n=277 GPs, n=219 community pharmacists)

**Fig. S4**: Usefulness of receiving important medication safety related information via several methods simultaneously (n=277 GPs, n=219 community pharmacists)

*Other (GPs) GP meeting, depends on alert/medication, medication file, informal discussion,

*Other (Community pharmacists) depends on alert/medication

**Figure S5:** Implementation of DHPC after it is opened (n=277 GPs, n=219 community pharmacists)

*Other: Had stopped prescribing prior to DHPC

**Fig. S6**: Actions taken by GPs following the most recent Irish DHPC on quinolone and fluoroquinolone prescribing issued in June 2023 (n=277 GPs, n=219 Community Pharmacists)

*Other for community pharmacists: discussed with local GPs, highlight side effect on patient information leaflet and attach to outside of box in dispensary

**Fig. S7**: Actions taken by pharmacists following the most recent Irish DHPC on quinolone and fluoroquinolone prescribing issued in June 2023 (n=277 GPs, n=219 community pharmacists)

*Other (GP): practice audit, ensured no woman prescribed it, was not practicing at the time

*Other (Community pharmacists): dispensary modifications (i.e. stickers/cards beside product, designated shelf, taped shelf edge alert), was not practicing at the time, audit,

Head office communication

**Fig. S8**: Actions taken following the most recent Irish DHPC issued on April 2018 on sodium valproate (n=277 GPs, n=219 community pharmacists)

Table S1: Comparison of self-reported recall of number of DHPCs received and the proportion acted upon in the last five years, by GPs (N=277) and community pharmacists (N=219).

|  | **GPs (N=277)** | | **Community Pharmacists (N=219)** | |
| --- | --- | --- | --- | --- |
| **Number of DHPCs** | **Received** | **Acted upon** | **Received** | **Acted upon** |
| 0-2 | 8 (3%) | 83 (30%) | 1 (1%) | 32 (15%) |
| 3-4 | 16 (6%) | 78 (28%) | 3 (1%) | 42 (19%) |
| 5-6 | 30 (11%) | 39 (14%) | 22 (10%) | 31 (14%) |
| 7-9 | 25 (9%) | 18 (7%) | 10 (5%) | 15 (7%) |
| 10 or more | 135 (49%) | 13 (5%) | 123 (56%) | 63 (29%) |
| Don’t know | 63 (23%) | 46 (17%) | 60 (27%) | 36 (16%) |
