# Supplementary material for: How direct healthcare professional communications are operationalised by general practitioners and community pharmacists in Ireland: a national cross sectional study
Source: Int J Clin Pharm. 2026 Mar 11;48(4):1303–14. doi: 10.1007/s11096-026-02105-3 (PMC13369712; doi:10.1007/s11096-026-02105-3)
Supplement: Supplementary file 2 — Supplementary file2 (DOCX 306 KB) [file 11096_2026_2105_MOESM2_ESM.docx]

***Pharmacist Questionnaire***

**Study title:** How are direct healthcare professional communications (DHPC) operationalised by GPs and community pharmacists in Ireland

Study information

Thank you for considering participating in this research project. The purpose of this document is to explain to you what this research is about and what your participation would involve, to enable you to make an informed choice. This is a survey, which will take up to**10 minutes** to complete.

The purpose of this study is to explore how Direct Healthcare Professional Communications (DHPCs), also known as ‘Dear Doctor/Pharmacist’ letters regarding medication safety are implemented in practice by general practitioners (GPs) and community pharmacists in Ireland. Should you choose to participate, you will be asked to complete an online survey, which will include questions on how the communications are implemented in practice.

Participation in this study is completely voluntary. There is no obligation to participate, and should you choose to do so you can refuse to answer specific questions or decide to withdraw from the study prior to completing the survey. All information you provide will be confidential and your anonymity will be protected throughout the study. Survey responses will be anonymous and no email IP addresses will be collected.

You maintain the right to withdraw from the study at any stage up to the point of survey submission. At this point, your data will be collated with that of other participants and can no longer be retrieved.

The anonymous data will be stored on the Department of General Practice UCC research server for minimum of ten years. The information you provide may contribute to research publications (i.e. peer reviewed journal) and/or conference presentations and a MD thesis.

On completion of this survey, there is an option to opt in to a 1-1 interview. Further details are provided at the end of the survey. This study has obtained ethical approval from the ICGP Research Ethics Committee.

If you have any queries about this research, you can contact me Dr Paul Ryan at [paul_ryan@ucc.ie](mailto:paul_ryan@ucc.ie), Mayfield Family Practice 021-4558850 or my supervisor Prof Emma Wallace, Professor of General Practice, UCC at [Ewallace@ucc.ie](mailto:Ewallace@ucc.ie)

If you agree to take part in this study, please complete the consent form below.

# Consent Form

Study title: How are direct health regulatory communications operationalised by GPs and community pharmacists in Ireland and what is their impact on prescribing?

**In order to proceed to the survey we require you to consent to the following. By checking the box for each statement, you are providing your consent.**

| I have read and understood the **Information Leaflet** about this research project. The information has been fully explained to me and I have been able to ask questions, all of which have been answered to my satisfaction. |  |
| --- | --- |
| I understand that I don’t have to take part in this study and that I can opt out at any time up until the point where I submit my responses. |  |
| I am aware of the potential risks, benefits and alternatives of this research study. |  |
| I have been assured that information about me will be kept private and confidential. |  |
| I have been given a copy of the Information Leaflet for my records. |  |
| I consent to take part in this research study having been fully informed of the risks, benefits, and alternatives. |  |
| I give informed explicit consent to have my data processed as part of this research study. |  |

**Survey**

**A. Demographics**

1. **Do you work in a community pharmacy?**

- **Yes**
- **No**

1. **How old (in years) are you?**

| **Age group** |  |
| --- | --- |
| 20-25 |  |
| 25-34 |  |
| 35-44 |  |
| 45-54 |  |
| 55-64 |  |
| 65-74 |  |
| 75-84 or over |  |

1. **Please tick the box that represents your gender**

| Gender |  |
| --- | --- |
| Male |  |
| Female |  |
| Non- binary |  |
| Prefer not to say |  |

1. **Please tick the box that represents how many years since you qualified as a pharmacist**

| <5 |  |
| --- | --- |
| 5-14 |  |
| 15-29 |  |
| >30 |  |
| Pharmacy Intern |  |

1. **State your primary workplace**

| Community Pharmacy* |  |
| --- | --- |
| Hospital |  |
| Academia |  |
| Industry |  |
| Other (please state) |  |

1. **In what type of pharmacy do you work?**

A single independent pharmacy

A small chain (2-10 pharmacies)

A large chain (more than 10 pharmacies)

N/A

1. **Are you a:**

| **Superintendent Pharmacist** |  |
| --- | --- |
| **Supervising Pharmacist** |  |
| **Support Pharmacist** |  |
| **Locum** |  |

1. **Please tick the box that represents how many years you have worked in community pharmacy since qualification.**

| <5 |  |
| --- | --- |
| 5-14 |  |
| 15-29 |  |
| ≥30 |  |
| Pharmacy Intern |  |

1. **Please tick the box that best represents the number of days a week you currently work in community pharmacy**

| 1 day or less |  |
| --- | --- |
| 2 to 3 days |  |
| 3 to 4 days |  |
| 4 to 5 days |  |
| >5 days |  |
| Not working in community pharmacy |  |

1. **Please tick the box that best represents the number of other pharmacists working in your pharmacy.**

| Solo Pharmacist |  |
| --- | --- |
| 1-2 |  |
| 2-4 |  |
| 5 or more |  |
| Not working in pharmacy practice |  |

1. **Please tick the box that best represents your site of work**

| I work in one site only |  |
| --- | --- |
| I work across multiple sites on a regular basis (i.e. Monday, Friday and Saturday in location A, Wednesday in location B) |  |
| I work in multiple sites on an irregular bases (Monday in site A, Tuesday in site B, Thursday in site C, Saturday in site D) |  |

1. **Please tick the box that best represents your pharmacy location**

| Rural (Town with a population of less than 1,500 persons) |  |
| --- | --- |
| Urban (Town with a population of greater than 1,500 persons) |  |
| Work in both rural and urban pharmacies regularly |  |
| Not working in community pharmacy |  |

**B. Medicine safety information experiences, beliefs, and perceptions**

1. **Where do you usually receive information on new important medicine safety updates? (You may select more than one option, if applicable)**

a) Direct Healthcare Professional Communication (DHPC from pharmaceutical company approved by the HPRA – also known as Dear Pharmacist letter)

b) Communication from HSE

c) Drug Safety Newsletter from HPRA

d) Continuing professional development activity

e) Work colleague(s)

f) Professional society

g) Patient(s) prescribed the drug

h) Other

1. **How are Direct healthcare professional communications (DHPCs) (i.e. letters from a pharmaceutical company and approved by the HPRA – also known as Dear Pharmacist letters) received by you in your pharmacy?**
   1. Email
   2. Letter
   3. Both email and letter
   4. I do not receive them
   5. Unsure/don’t know
2. **What is your preferred method of correspondence regarding important medicine safety related information ?**
   1. Email
   2. Letter
   3. Both
   4. Other-please specify

**14 (a) Approximately how many of these Dear Pharmacist letters regarding medication safety (from a pharmaceutical company & the HPRA) did you receive in the last five years?**

a. 0-2

b. 3-4

c. 5-6

d. 7-9

e. 10 or more

f. Don’t know

**14b Please estimate the number of these Dear Pharmacist letters you implemented action as a result of (e.g. informing colleagues, discuss with patient etc) in the last five years?**

a. 0-2

b. 3-4

c. 5-6

d. 7-9

e. 10 or more

f. Don’t know

1. **How useful do you think DHPCs (i.e. Dear Pharmacist letters) are in relation to safe prescribing and dispensing of medicines are?**
2. Not at all useful
3. Slightly useful
4. Moderately useful
5. Very useful
6. Extremely useful
7. **In what format would you prefer to receive correspondence about important medicine safety related information ? Rank in order of preference**

a) Dear Pharmacist letter

b) Dear Pharmacist email

c Both DHPC by letter AND email

d) HPRA website

e) Publications (i.e., Pharmacy magazines and other publications)

f) Professional organization (e.g., IIOP) email communication

g) Professional organization webinar

h) Other, please specify

1. **How useful would you find receiving important medicine safety related information via several methods simultaneously (i.e., by post and by email)?**
2. Not at all useful
3. Slightly useful
4. Moderately useful
5. Very useful
6. Extremely useful
7. **How useful do you consider repeated alerts regarding important medicine safety related information to be (i.e. repetition of the letter or email on several occasions over a period of time)?**
8. Not at all useful
9. Slightly useful
10. Moderately useful
11. Very useful
12. Extremely useful

**C. Implementation in practice**

1. What is the main safety issue associated with Sodium Valproate (Epilim^®^) which led to a DHPC (i.e. ‘Dear Pharmacist’) letter in April 2018
   1. Tendonitis
   2. Vision problems
   3. Gastric Ulceration
   4. Teratogenicity
   5. None of the above
   6. All of the above
2. **What actions did you take following the most recent DHPC (i.e. Dear Pharmacist letter) issued on April 2018 on sodium valproate?**

a) We had a pharmacy meeting to discuss

b) I discussed this with my pharmacist colleagues

c) We discussed this at our local continuing professional development meeting

d)We identified relevant patients and put a note on their file

e) I was more aware when dispensing Valproate and was more likely to counsel patients on these adverse effects

f) We discussed this with our patients when they requested their prescription

e)No action taken

f)Other – free type option if this is chosen

1. **What are the main safety issues associated with quinolone and fluoroquinolone antibiotics (e.g. ciprofloxacin (Ciproxin^®^), levofloxacin (Tavanic^®^), moxifloxacin (Avelox^®^)which led to a recent DHPC (i.e. ‘Dear Pharmacist’ letter)?**
   1. Disabling, long-lasting and potentially irreversible adverse reactions mainly affecting musculoskeletal and nervous systems.
   2. Gastric Ulceration and teratogenicity
   3. Weight decrease and hypernatraemia
   4. Hyperthyroidism and Constipation
   5. All of the above
   6. None of the above
2. **What actions did you take following the most recent DHPC (i.e. Dear Pharmacist letter) on quinolone and fluoroquinolone prescribing issued in June 2023? Tick all that apply**

a) We had a pharmacy meeting to discuss

d) We discussed it with our patients when they requested their prescription

e) I was more aware of the adverse effects of quinolones

f) Discussed at local continuing professional development meeting

e) No action taken

f) Other – free type option if this is chosen

1. **Who opens the DHPC (i.e. Dear Pharmacist letter) after it arrives in your email inbox/post in your pharmacy?**
   1. Pharmacy technician
   2. Over the counter staff
   3. Pharmacist
   4. Supervising pharmacist
   5. Don’t know
   6. Other – please specify
2. **After the DHPC (i.e. Dear Pharmacist letter) is opened, how is the communication implemented in the pharmacy? Tick all which apply**

a) Dear Pharmacist letter/email left in a post tray for the pharmacist(s) to read

b) Relevant patients are identified and contacted

c) Alerts are put on relevant patients file to discuss next time patient is in to collect a prescription

d) Discussed at the pharmacy staff meeting

e) Used to inform pharmacy audit topics

f) Discussed at local continuing professional development meeting

g) Communicated to local GP colleagues (i.e. by phone call, via health mail)

f) Pharmacy Whatsapp group

g)Put in the handover diary for pharmacist

g) Don’t know

f) Other – please specify

1. **How is the DHPC (i.e. Dear Pharmacist letter) disseminated to pharmacists and technicians in your pharmacy?**
   1. It is photocopied and shared in the trays of all pharmacists
   2. It is photocopied and shared in the trays of all pharmacists and pharmacy technicians
   3. Internal pharmacy email communication
   4. Each pharmacist already receives the communication directly, so we do not disseminate
   5. Pharmacy meeting
   6. Pharmacy staff Whatsapp group
   7. Other – please specify
2. **If pharmacist or other colleagues are on leave (e.g., annual leave, maternity leave etc) how is the information from the DHPC (i.e. Dear Pharmacist letter) communicated to them?**

a) Their pharmacy tray

b) Internal pharmacy email

c) Pharmacy staff WhatsApp group

d) Other – please specify

1. **Is the information from the DHPC (i.e. Dear Pharmacist letter) communicated to new staff/ pharmacy locums?**

Yes

No

Other -please specify

1. If the DHPC (i.e. Dear Pharmacist letter) is communicated to new staff/pharmacy locums how is this done?
   1. Not applicable as not communicated to new staff/pharmacy locums
   2. Flagged on patient file
   3. Left in pharmacist tray
   4. Handover diary
   5. Communicated by phone/message
   6. Other – please specify
2. **Are there any barriers that prevent you from implementing DHPC (i.e. Dear Pharmacist letter) recommendations in your pharmacy?**

a) Unsure if any changes are required

b) Unsure who is responsible for making changes

c) Time constraints

d) Lack of pharmacy team meetings

e) No staff email/way to communicate with staff

f) Lack of prescribing alert notifications from software system (i.e. Mclernons, touchstore)

g) No barriers identified

g) Other – free text

1. **All dispensing software systems should give significant medication safety notifications to alert the pharmacist when a medication associated with a HPRA related safety alert is dispensed i.e. teratogenic potential with Epilim**

Yes

No

Don’t know

1. **Would you be willing to receive remote support from a pharmacist or GP colleague to help you implement recommended changes as part of DHPC (i.e. Dear Pharmacist letters)?**

Yes

No

Don’t know

**Thank you for completing this survey! If you have any comments or queries please contact** [paul_ryan@ucc.ie](mailto:paul_ryan@ucc.ie)

************************************************************************

**We would now like to invite you to participate in an optional additional study which involves a one-to-one interview online with Dr Paul Ryan.**

These interviews will expand on the overall aggregated survey results across three main domains (process on how DHPC i.e. Dear Pharmacist letters are implemented in practice, facilitators, and barriers to implementation) with the overall aim of finding out how these communications are currently implemented and how or if this can be improved.

These interviews (approx. 30 minutes duration) will be conducted online via Microsoft Teams with Dr Paul Ryan, GP, Mayfield Cork & Pharmacist.

If you are interested in participating, please contact Dr Paul Ryan via email [paul_ryan@ucc.ie](mailto:paul_ryan@ucc.ie) for further details.

***GP Questionnaire***

**Study title:** How are direct healthcare professional communications (DHPC) operationalised by GPs and community pharmacists in Ireland

Study information

Thank you for considering participating in this research project. The purpose of this document is to explain to you what this research is about and what your participation would involve, to enable you to make an informed choice. This is a survey, which will take up to**10 minutes** to complete.

The purpose of this study is to explore how Direct Healthcare Professional Communications (DHPCs), also known as dear Doctor/Pharmacist letters regarding medication safety are implemented in practice by general practitioners (GPs) and community pharmacists in Ireland. Should you choose to participate, you will be asked to complete an online survey, which will include questions on how the communications are implemented in practice.

Participation in this study is completely voluntary. There is no obligation to participate, and should you choose to do so you can refuse to answer specific questions or decide to withdraw from the study prior to completing the survey. All information you provide will be confidential and your anonymity will be protected throughout the study. Survey responses will be anonymous and no email IP addresses will be collected.

You maintain the right to withdraw from the study at any stage up to the point of survey submission. At this point, your data will be collated with that of other participants and can no longer be retrieved.

The anonymous data will be stored on the Department of General Practice UCC research server for minimum of ten years. The information you provide may contribute to research publications (i.e. peer reviewed journal) and/or conference presentations and a MD thesis.

On completion of this survey, there is an option to opt in to a 1-1 interview. Further details are provided at the end of the survey. This study has obtained ethical approval from the ICGP Research Ethics Committee.

If you have any queries about this research, you can contact me Dr Paul Ryan at [paul_ryan@ucc.ie](mailto:paul_ryan@ucc.ie), Mayfield Family Practice 021-4558850 or my supervisor Prof Emma Wallace, Professor of General Practice, UCC at [Ewallace@ucc.ie](mailto:Ewallace@ucc.ie)

If you agree to take part in this study, please complete the consent form below.

# Consent Form

Study title: How are direct health regulatory communications operationalised by GPs and community pharmacists in Ireland and what is their impact on prescribing?

**In order to proceed to the survey we require you to consent to the following. By checking the box for each statement, you are providing your consent.**

| I have read and understood the **Information Leaflet** about this research project. The information has been fully explained to me and I have been able to ask questions, all of which have been answered to my satisfaction. |  |
| --- | --- |
| I understand that I don’t have to take part in this study and that I can opt out at any time up until the point where I submit my responses. |  |
| I am aware of the potential risks, benefits and alternatives of this research study. |  |
| I have been assured that information about me will be kept private and confidential. |  |
| I have been given a copy of the Information Leaflet for my records. |  |
| I consent to take part in this research study having been fully informed of the risks, benefits, and alternatives. |  |
| I give informed explicit consent to have my data processed as part of this research study. |  |

**Survey**

1. **How old (in years) are you?**

| Age group |  |
| --- | --- |
| 20-25 |  |
| 25-34 |  |
| 35-44 |  |
| 45-54 |  |
| 55-64 |  |
| 65-74 |  |
| 75-84 or over |  |

1. **Please tick the box that represents your gender**

| Gender |  |
| --- | --- |
| Male |  |
| Female |  |
| Non- binary |  |
| Prefer not to say |  |

1. **Please tick the box that represents how many years since you qualified as a GP**

| <5 |  |
| --- | --- |
| 5-14 |  |
| 15-29 |  |
| ≥30 |  |

1. **State your primary workplace**

| General Practice |  |
| --- | --- |
| Academia |  |
| Other (please state) |  |

1. **Please tick the box that represents how many years you have worked in General Practice**

| <5 |  |
| --- | --- |
| 5-14 |  |
| 15-29 |  |
| ≥30 |  |

1. **Are you employed as a:**

| GP Principal |  |
| --- | --- |
| Single handed GP |  |
| Salaried GP |  |
| Assistant GP |  |
| Sessional GP |  |
| Locum GP |  |
| Other |  |

1. **Please tick the box that best represents the number of days a week you work in General Practice**

| 1 day or less |  |
| --- | --- |
| 2 to 3 days |  |
| 4 to 5 days |  |
| >5 days |  |
| Not working in clinical practice |  |

1. **Please tick the box that best represents the number of other GPs working with you**

| 0 (Single handed) |  |
| --- | --- |
| 1-2 |  |
| 2-4 |  |
| 5 or more |  |
| Not working in clinical practice |  |

1. **Please tick the box that best represents your daytime site of work**

| I work in one site only |  |
| --- | --- |
| I work across multiple sites on a regular basis (i.e. Monday, Friday and Saturday in location A, Wednesday in location B) |  |
| I work in multiple sites on an irregular bases (Monday in site A, Tuesday in site B, Thursday in site C, Saturday in site D) |  |

1. **Please tick the box that best represents your practice location**

| Rural (Town with a population of less than 1,500 persons) |  |
| --- | --- |
| Urban (Town with a population of greater than 1,500 persons) |  |
| Locum |  |
| Work in both rural and urban General Practices regularly |  |
| Not working clinically |  |

**B. Medicine safety information experiences, beliefs, and perceptions**

1. **Where do you usually receive information on new important medicine safety updates? (You may select more than one option, if applicable)**

a) Direct Healthcare Professional Communication (DHPC from pharmaceutical company approved by the HPRA – also known as Dear Doctor letter)

b) Communication from HSE

c) Drug Safety Newsletter from HPRA

d) Continuing professional development activity

e) Work colleague(s)

f) Professional society e.g. ICGP

g) Patient(s) prescribed the drug

h) Other

1. **How are Direct healthcare professional communications (DHPCs) (i.e. letters from a pharmaceutical company and approved by the HPRA – also known as Dear Doctor letters) received by you in your general practice?**
   1. Email
   2. Letter
   3. Both email and letter
   4. I do not receive them
   5. Unsure/don’t know
2. **What is your preferred method of correspondence regarding important medicine safety related information ?**
   1. Email
   2. Letter
   3. Both
   4. Other-please specify

**14. (a) Approximately how many of these Dear Doctor letters regarding medication safety (from a pharmaceutical company & the HPRA) did you receive in the last five years?**

a. 0-2

b. 3-4

c. 5-6

d. 7-9

e. 10 or more

f. Don’t know

14.b **Please estimate the number of these Dear Doctor letters you implemented action as a result of (e.g. informing colleagues, discuss with patient etc) in the last FIVE years?**

a. 0-2

b. 3-4

c. 5-6

d. 7-9

e. 10 or more

f. Don’t know

**15. How useful do you think DHPCs (i.e. Dear Doctor letters) in relation to safe prescribing and dispensing of medicines are?**

1. Not at all useful
2. Slightly useful
3. Moderately useful
4. Very useful
5. Extremely useful
6. **In what format would you prefer to receive correspondence about important medicine safety related information ?**

a) DHPC by letter

b) DHPC by email

c) Both DHPC by letter AND email

d) HPRA website

e) Publications (i.e. GP Forum and other publications)

f) Professional organization (e.g. ICGP) email communication

g) Professional organization (e.g. ICGP) webinar

h) Other, please specify

1. **How useful would you find receiving important medicine safety related information via several methods simultaneously (i.e., by post and by email)?**
2. Not at all useful
3. Slightly useful
4. Moderately useful
5. Very useful
6. Extremely useful
7. **How useful do you consider repeated alerts regarding important medicine safety related information to be (i.e. repetition of the letter or email on several occasions over a period of time)?**
8. Not at all useful
9. Slightly useful
10. Moderately useful
11. Very useful
12. Extremely useful

**C. Implementation in practice**

1. What is the main safety issue associated with Sodium Valproate (Epilim^®^) which led to a DHPC (i.e. ‘Dear Doctor’) letter in April 2018
   1. Tendonitis
   2. Vision problems
   3. Gastric Ulceration
   4. Teratogenicity
   5. None of the above
   6. All of the above
2. **What actions did you take following the most recent DHPC (i.e. Dear Doctor letter) issued on April 2018 on sodium valproate?**

a) We had a practice meeting to discuss

b) We identified relevant patients and put a note on their file

c) We discussed this at our local CME meeting

d) I was more aware when prescribing sodium valproate and was more likely to counsel patients on these adverse effects

d) We discussed it with our patients when they requested their prescription

e) We identified relevant patients and called them in to discuss

f) Other – free type option if this is chosen

g) No action taken

1. **What are the main safety issues associated with quinolone and fluoroquinolone antibiotics (e.g. ciprofloxacin (Ciproxin^®^), levofloxacin (Tavanic^®^), moxifloxacin (Avelox^®^)which led to a recent DHPC (i.e. ‘Dear Doctor’ letter)?**
   1. Disabling, long-lasting and potentially irreversible adverse reactions mainly affecting musculoskeletal and nervous systems.
   2. Gastric Ulceration and teratogenicity
   3. Weight decrease and hypernatraemia
   4. Hyperthyroidism and Constipation
   5. All of the above
   6. None of the above
2. **What actions did you take following the most recent DHPC (i.e. Dear Doctor letter) on quinolone and fluoroquinolone prescribing issued in June 2023? Tick all that apply.**

a) I have reduced my prescribing of quinolones

b) I did not change my prescribing of quinolones

c) We had a practice meeting to discuss

d) We discussed this at our local CME group

e) I was more aware of my prescribing of quinolones

f) We did an audit on our prescribing of quinolones

g) Other – free type option if this is chosen

1. **Who opens the DHPC (i.e. Dear Doctor letter) after it arrives in your email inbox/post in your practice?**
   1. Administrative staff
   2. Practice manager
   3. Practice nurse
   4. GP Principal
   5. Salaried GP
   6. Other – please specify
2. **After the DHPC (i.e. Dear Doctor letter) is opened, how is the communication implemented in the practice? Tick all which apply**

a) Dear Doctor letter/email left in a tray for the GP(s) to read

b) Relevant patients are identified and contacted

c) Alerts are put on relevant patients file to discuss next time patient requests a prescription

d) Discussed at the practice staff meeting

e) Used to inform general practice audit topics

f) Discussed at local continuing medical education meeting

g) Communicated to pharmacy colleagues (i.e. by phone call, via health mail)

h) Other – please specify

1. **How is the DHPC (i.e. Dear Doctor letter) disseminated to practice staff?**
   1. It is photocopied and shared in the trays of all GPs
   2. It is photocopied and shared in the trays of all GP clinical staff e.g. GPs, practice nurses
   3. Internal GP email communication
   4. Each GP already receives the communication directly, so we do not disseminate
   5. Practice team meeting
   6. GP practice Whatsapp group
   7. Don’t know
   8. Other – please specify
2. **If a GP or other colleagues are on leave (e.g., annual leave, maternity leave etc) how is the information from the DHPC (i.e Dear Doctor letter) communicated to them?**

a) Their GP post tray

b) Internal practice email

c) GP practice WhatsApp group

d) Other – please specify

1. **Is the information from the DHPC (i.e. Dear Doctor letter) communicated to new staff/ GP locums?**

Yes

No

Other -please specify

Follow on question if clicked Yes:

‘How is it communicated i.e. flagged on patient file/left in GP tray/Handover diary/SMS messaging i.e. Whatsapp/Other -please specify

1. **Are there any barriers that prevent you from implementing DHPC (i.e. Dear Doctor letter) recommendations in your GP practice?**

a) Unsure if any changes are required

b) Unsure who is responsible for making changes

c) Time constraints

d) Lack of practice team meetings

e) No staff email/way to communicate with staff

f) Lack of notifications from practice software (i.e. Socrates/Helix Health)

g) No barriers identified

h) Other – free text

1. **All prescribing software systems should give significant medication safety notifications to alert the GP when a medication associated with a HPRA related safety alert is prescribed i.e. teratogenic potential with Epilim**

Yes

No

Don’t know

1. **Would you be willing to receive remote prescribing support from a pharmacist or GP colleague to help you implement recommended changes as part of DHPC (i.e. Dear Doctor letters)?**

Yes

No

Don’t know

**Thank you for completing this survey!**

************************************************************************

**We would now like to invite you to participate in an optional additional study which involves a one-to-one interview online with Dr Paul Ryan.**

These interviews will expand on the overall aggregated survey results across three main domains (process on how DHPC i.e. Dear Doctor letters are implemented in practice, facilitators, and barriers to implementation) with the overall aim of finding out how these communications are currently implemented and how or if this can be improved.

These interviews (approx. 30 minutes duration) will be conducted online via Microsoft Teams with Dr Paul Ryan, GP, Mayfield Cork & Pharmacist.

If you are interested in participating, please contact Dr Paul Ryan via email [paul_ryan@ucc.ie](mailto:paul_ryan@ucc.ie) for further details.
